# Supplementary material for: Understanding CRY2 interactions for optical control of intracellular signaling
Source: Nat Commun. 2017 Sep 15;8:547. doi: 10.1038/s41467-017-00648-8 (PMC5601944; doi:10.1038/s41467-017-00648-8)
Supplement: Supplementary file 1 — Supplementary Information [file 41467_2017_648_MOESM1_ESM.pdf]

Description of Supplementary Files

File Name: Supplementary Information
Description: Supplementary Figures, Supplementary Table and Supplementary Methods

File Name: Supplementary Movie 1
Description: **Cytosolic CRY2wt, CRY2(neutral2-6) or CRY2(Δ2-6) was recruited to the ER-bound CIB1 and oligomerized into clusters under light stimulation.** Shown are the time-lapse images of COS7 cells expressing CIB1-GFP-Sec61 along with mCh-tagged CRY2wt, CRY2(neutral2-6) or CRY2(Δ2-6) respectively. Blue light pulses were delivered at 2 s intervals for 100 s.

File Name: Supplementary Movie 2
Description: **CRY2(E490G) and CRY2(Δ490-498) formed visible clusters under light stimulation while CRYwt, CRY2(neutral2-6), CRY2(Δ2-6), and other C-terminal truncated CRY2 mutants did not.** Shown are the time-lapse images of COS7 cells expressing mCh-tagged CRY2wt or CRY2 mutants respectively. Blue light pulses were delivered at 5 s intervals for 500 s.

File Name: Supplementary Movie 3
Description: **CRY2(R489D) and CRY2(R489E) did not form noticeable clusters under light stimulation while other CRY2 derivatives with 489 and/or 490 mutations aggregated drastically.** Shown are the time-lapse images of COS7 cells expressing each mCh-tagged CRY2 mutant with 489 and/or 490 mutations. Blue light pulses were delivered at 5 s intervals for 500 s.

File Name: Supplementary Movie 4
Description: **CRY2wt or CRY2 mutants with C-terminal negative charges was recruited to the ER-bound CIB1 and oligomerized into clusters to different extents under light stimulation.** Shown are the time-lapse images of COS7 cells expressing CIB1-GFP-Sec61 along with mCh-tagged CRY2wt and each CRY2 mutant with C-terminal negative charges respectively. Blue light pulses were delivered at 2 s intervals for 20 s.

File Name: Supplementary Movie 5
Description: **CRY2wt or CRY2low fused with mCh or tdTom was recruited to the ER-bound CIB1 and oligomerized into clusters to different extents under light stimulation.** Shown are the time-lapse images of COS7 cells expressing CIB1-GFP-Sec61 along with mCh or tdTom -tagged CRY2wt and CRY2low respectively. Blue light pulses was delivered at 2 s intervals for 20 s.

File Name: Peer Review File
Description:

Supplementary Figures

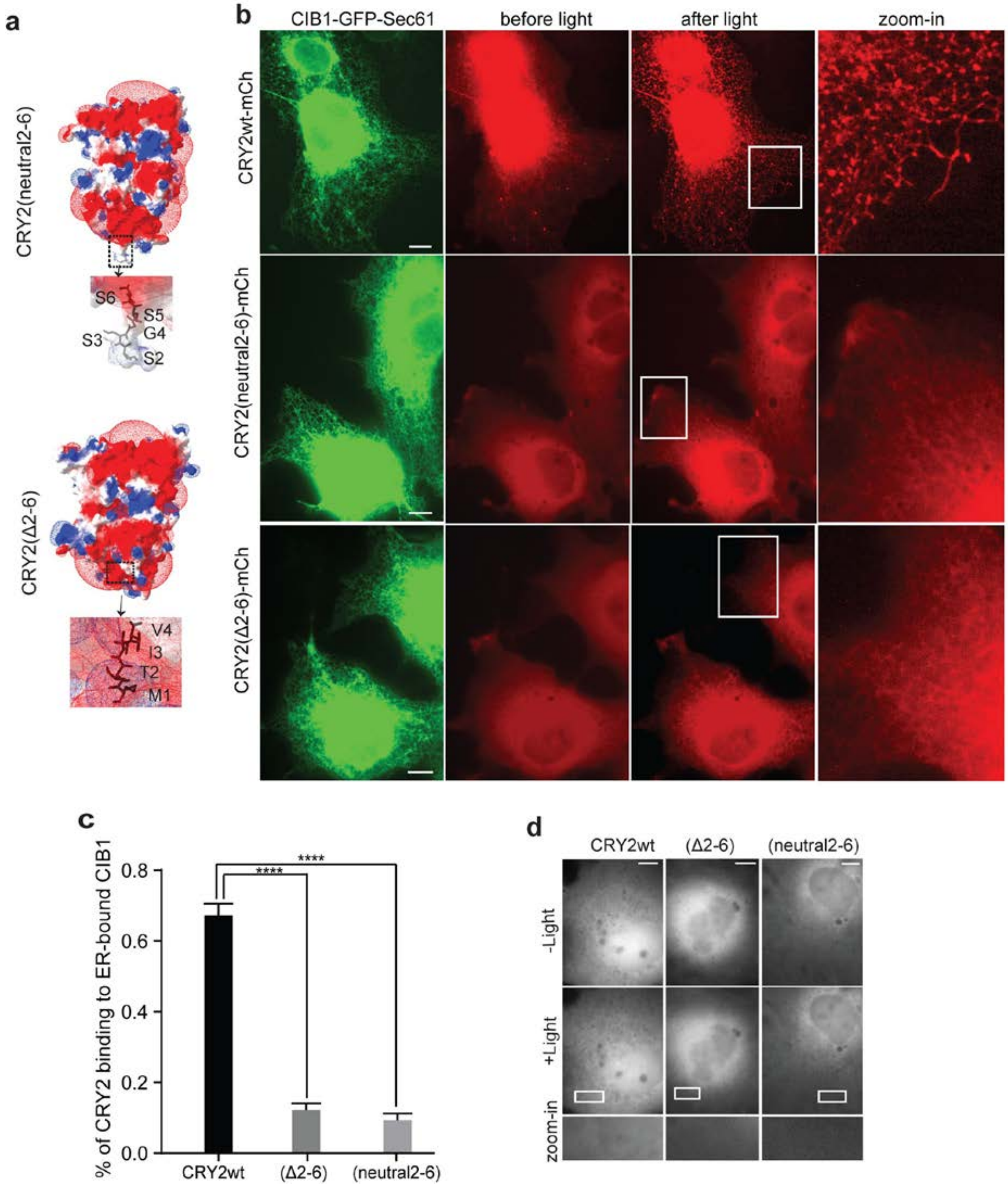

Supplementary Figure 1. CRY2(neutral2-6) or CRY2(Δ2-6) dimerized with ER-bound CIB1 much less than CRY2wt, indicating the affected CIB1-binding capacity. (**a**) The electrostatic potential maps for CRY2(neutral2-6) or CRY2(Δ2-6) showing that the positive charges at the N-terminus are removed. (**b**) The light-induced CRY2-CIB1 binding is much

weaker for CRY2(neutral2-6) or CRY2(Δ2-6), as compared to CRY2wt. One pulse of 200-ms blue light was delivered to COS7 cells co-transfected with CIB1-GFP-Sec61 and each mCh-tagged CRY2 respectively. Multiple cells were included in the images in the same field of view. (**c**) COS7 cells were co-transfected with CIB1-GFP-Sec61 and each mCh-tagged CRY2 respectively and exposed to one 200 ms pulse of blue light. The average mCh intensities before light stimulation (I_{dark}) or after light stimulation (I_{light}) were measured by ImageJ in a selected cytosolic area in the COS7 cell. $I_{background}$ is the image background. The percentage of light-induced CRY2 binding to ER-bound CIB1 was calculated by %binding = 1- $(I_{light} - I_{background}) / (I_{dark} - I_{background})$. For each COS7 cell, 5 different cytosolic areas were randomly selected and measured. The CRY2 binding affinity to CIB1 in each cell was indicated by the average of the CRY2 binding percentages in these 5 different locations. For each transfection condition, 10 cells were measured and results are presented as means ± s.e.m. (n=10,10,10). Results were analyzed using one-way ANOVA with Dunnett's post hoc test (****P=0.0001). (**d**) Cytosolic CRY2(neutral2-6) or CRY2(Δ2-6) did not form clusters upon blue light stimulation, similar to CRY2wt. COS7 cells were transfected with mCh-tagged CRY2 and stimulated with blue light at 5 s intervals for 500 s. Scale bars, 10µm.

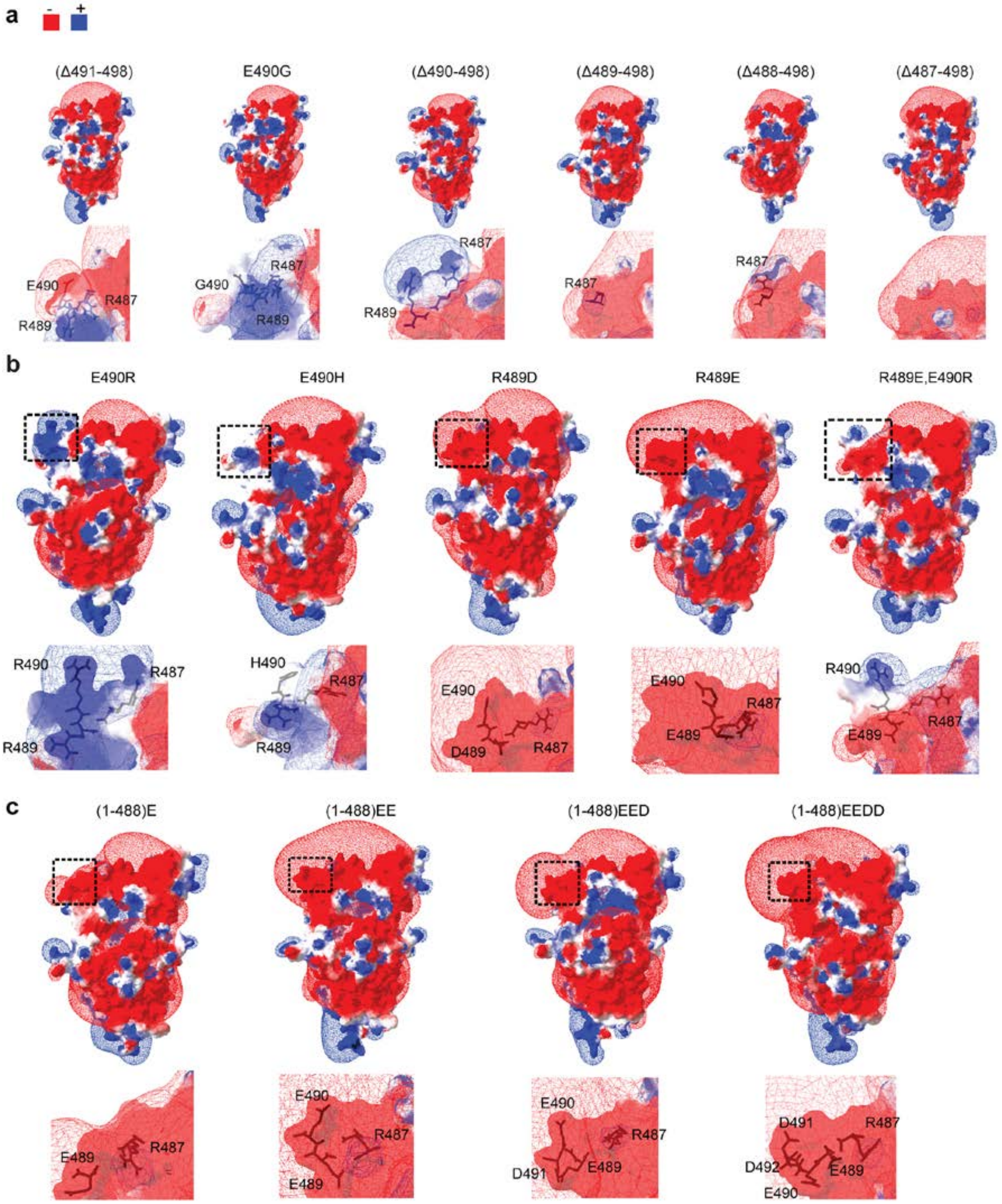

Supplementary Figure 2. The electrostatic potential maps for CRY2 mutants. (a) Surface charge maps for CRY2 with C-terminal truncations and E490G mutation. (b) Surface charge maps for CRY2 mutations at positions 489 and 490. The bottom panel shows the insets of CRY2 C-terminus indicated by black dashed boxes. (c) Surface charge maps for truncated CRY2s with increasing number of negative charges at the C-terminus. The bottom panel shows the insets of CRY2 C-terminus indicated by black dashed boxes.

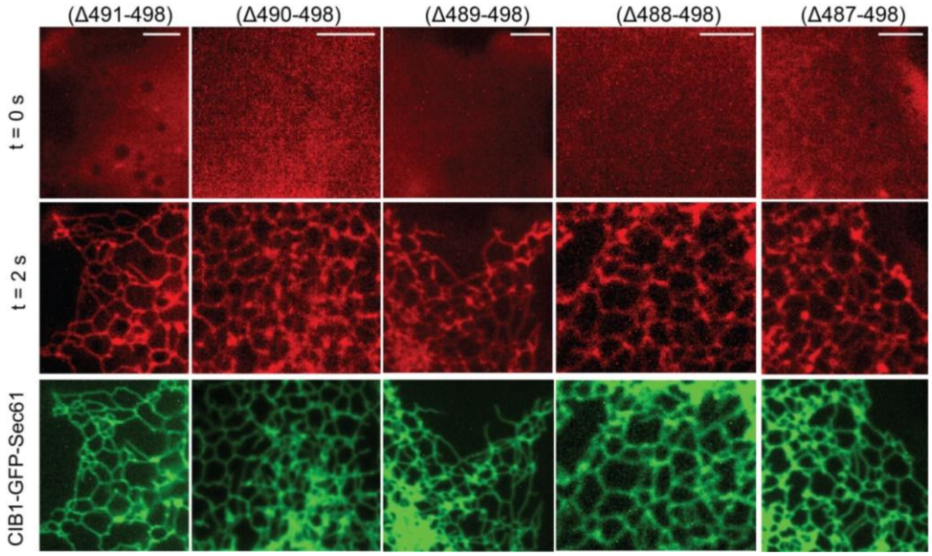

Supplementary Figure 3. C-terminal truncated mutants of CRY2 were completely recruited to the ER membrane via CRY2-CIB1 dimerization, indicating the preserved CIB1-binding ability. COS7 cells were co-transfected with CIB1-GFP-Sec61 and each truncated version of CRY2. Blue light was delivered at 2 s intervals for 100 s. Before light stimulation, mCh-conjugated CRY2 was distributed homogeneously. After one 200ms pulse of blue light, almost all CRY2 was recruited to the ER membrane. Scale bars, 5µm.

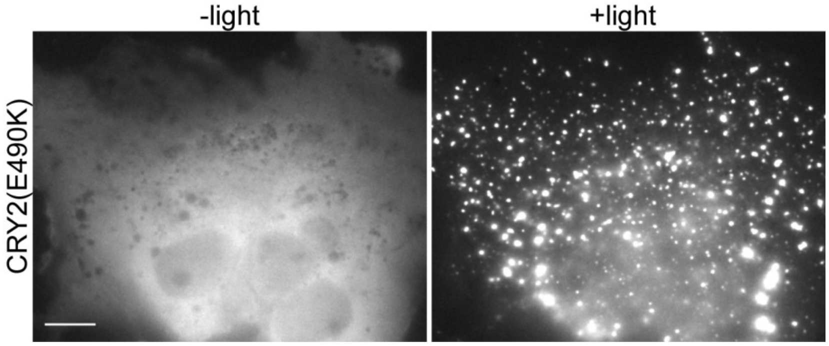

Supplementary Figure 4. CRY2(E490K) has enhanced clustering capacity. COS7 cells were transfected with CRY2(E490K)-mCh and subject to blue light illumination at 5 s intervals for a total of 500 s. Before any blue light stimulation, CRY2(E490K) distributed homogeneously across the cell. After light exposure, CRY2(E490K) aggregated drastically into clusters. Scale bar, 10 µm.

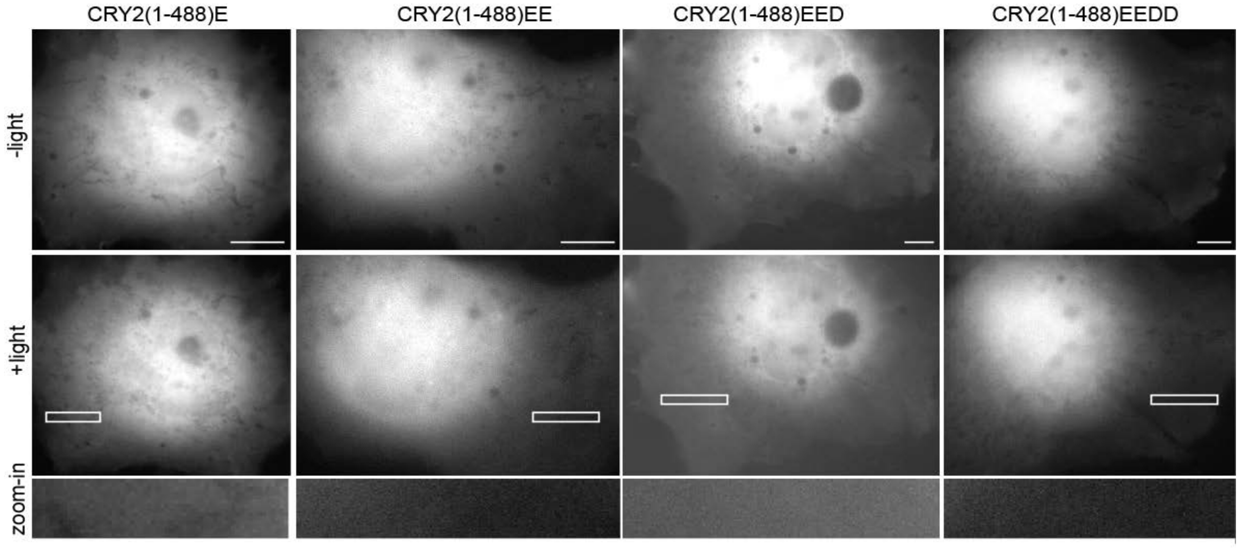

Supplementary Figure 5. CRY2 mutants with increasing numbers of negative charges at the C-terminus of CRY2(1-488) did not form noticeable clusters in cytosol. CRY2(1-488)E, CRY2(1-488)EE, CRY2(1-488)EED and CRY2(1-488)EEDD were tagged with mCh and expressed in COS7 cells respectively. Blue light was delivered at 5 s intervals for 500 s in total. None of the CRY2 derivatives formed visible clusters in the cytosol after light illumination. Scale bars, 10µm.

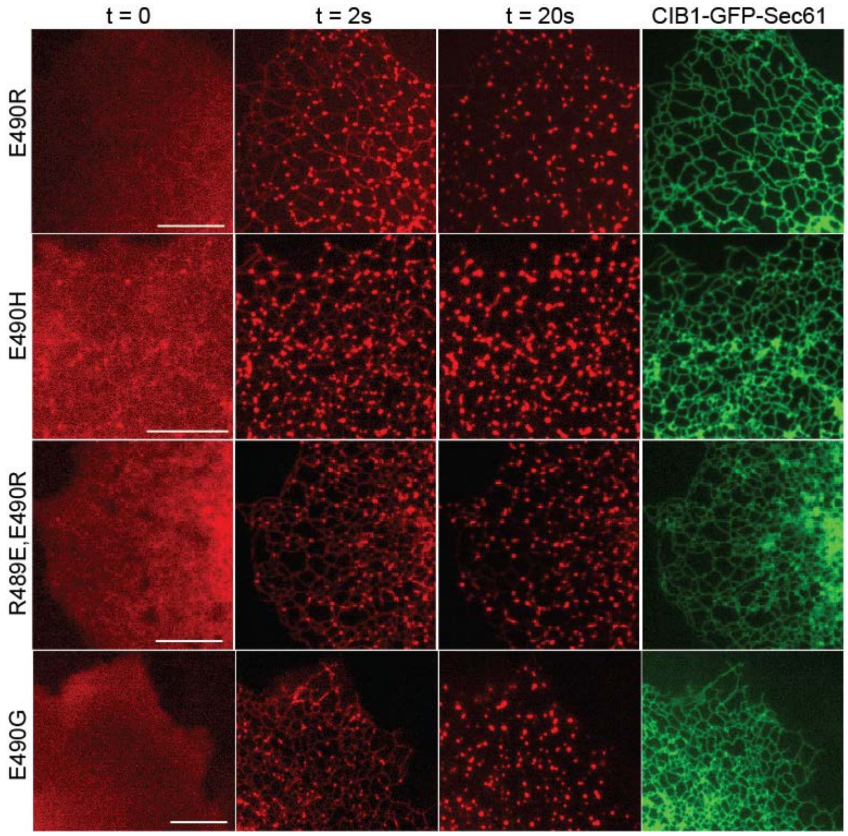

Supplementary Figure 6. CRY2 mutants (E490R, E490H, (R489E,E490R), E490G) were completely recruited to the ER membrane via CRY2-CIB1 dimerization, indicating the preserved CIB1-binding ability. COS7 cells were co-transfected with CIB1-GFP-Sec61 and each mCh-tagged CRY2 variants and exposed to blue light pulses at 2 s intervals. Prior to light stimulation, CRY2 was diffusively distributed in cells. Immediately after one pulse of blue light exposure, these CRY2 mutants were completely recruited to the ER membrane and showed clear clustering. After 20 s, CRY2 mutants clustered even more drastically. Scale bars, 10 µm.

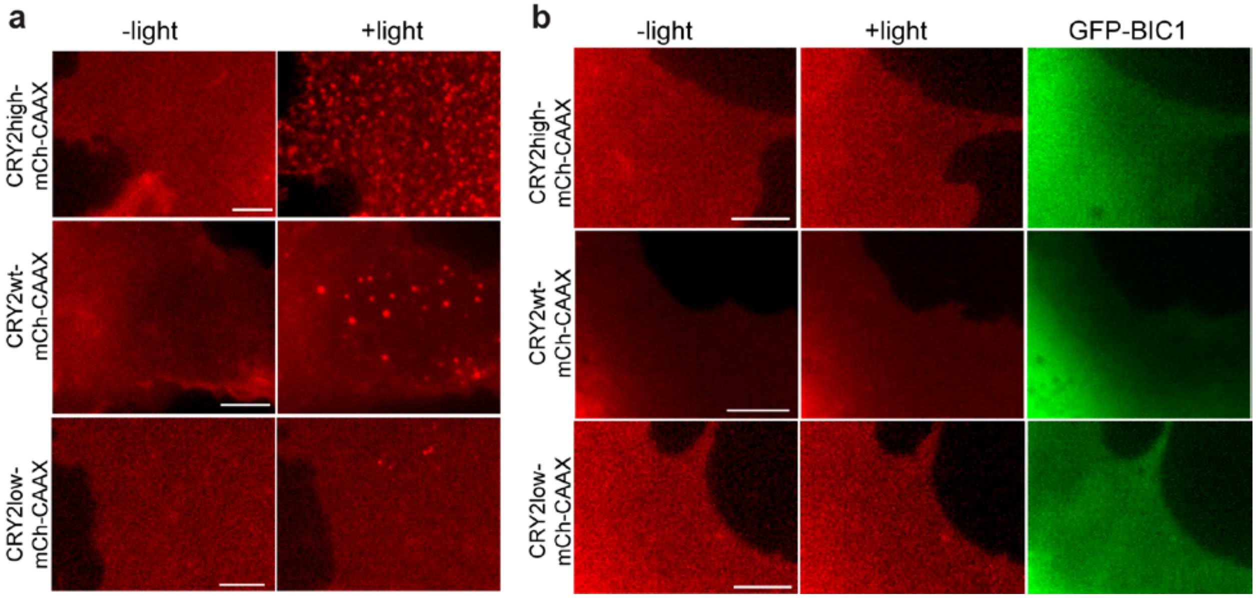

Supplementary Figure 7. BIC1 can inhibit light-mediated cluster formation of CRY2high, CRY2wt and CRY2low. COS7 cells were transfected with CRY2high-mCh-CAAX, CRY2wt-mCh-CAAX, and CRY2low-mCh-CAAX respectively (a) or with GFP-BIC1 (b). One pulse blue light of 100 ms exposure was delivered to COS7 cells and CRY2 distribution was acquired at t=0s and t=100 s. (**a**) The clusters formed increased drastically from CRY2low, CRY2wt and CRY2high. (**b**) In the presence of GFP-BIC1, none of CRY2low, CRY2wt and CRY2high formed any noticeable cluster. Scale bars, 5µm.

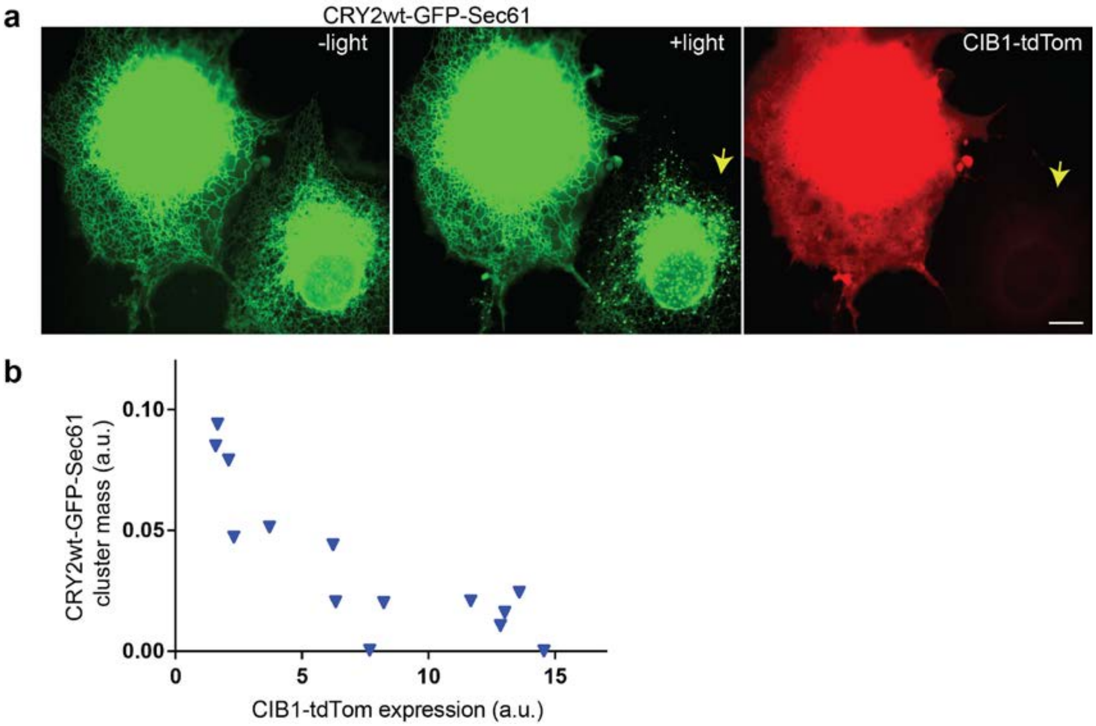

Supplementary Figure 8. tdTom-fused CIB1 suppresses CRY2 clustering in a concentration-dependent manner. Blue light was delivered to COS7 cells for 10 pulses at 5 s intervals. COS7 cells were co-transfected with CRY2-GFP-Sec61 and CIB1-tdTom. (**a**) CRY2 oligomerization was largely inhibited in the cells with higher expression of CIB1-tdTom (left cell) while not visibly suppressed in the cell with lower expression (right cell, indicated by a yellow arrow). (**b**) The masses of CRY2 cluster on the ER membrane in cells with different concentrations of CIB1-tdTom (n=14). Scale bar, 10µm.

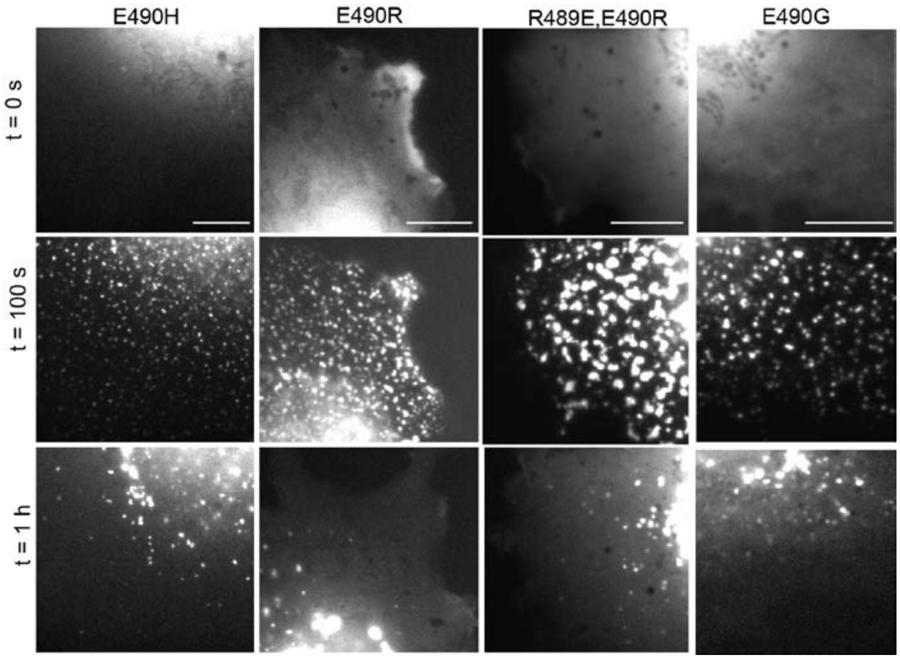

Supplementary Figure 9. Large clusters formed by CRY2 mutant exhibiting strong oligomerization stayed for at least one hour. One 1 s pulse of blue light was delivered to COS7 cells expressing CRY2(E490H), CRY2(E490R), CRY2(R489E, E490R) or CRY2(E490G). At t=100 s, they all formed a lot of clusters in the cytosol. After 1h without blue light, some of the large clusters remained. Scale bars, 5 µm.

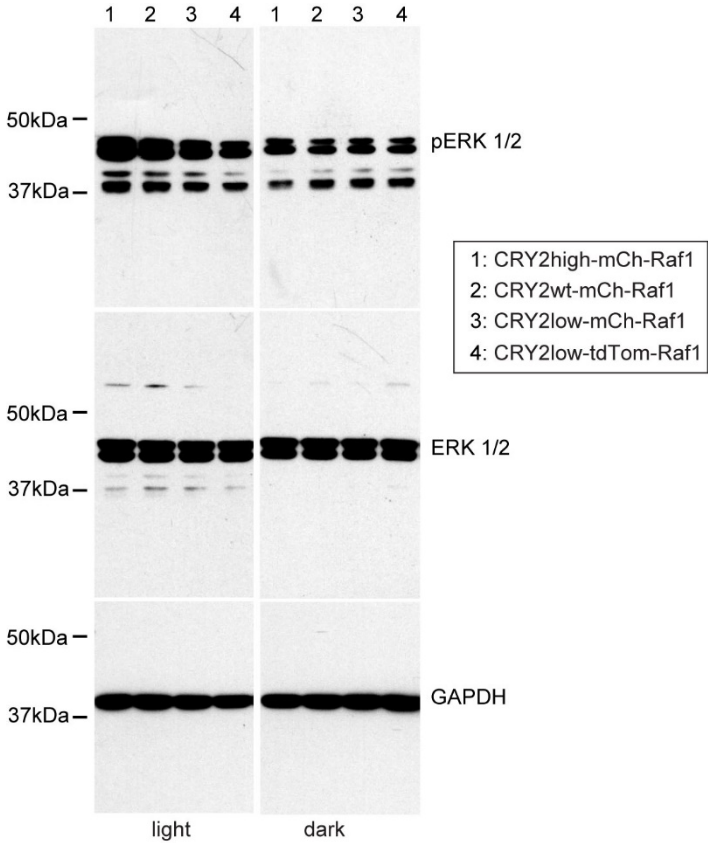

Supplementary Figure 10. Uncropped scans of western blots. The images in Fig. 5c are cropped from the full scans of blots.

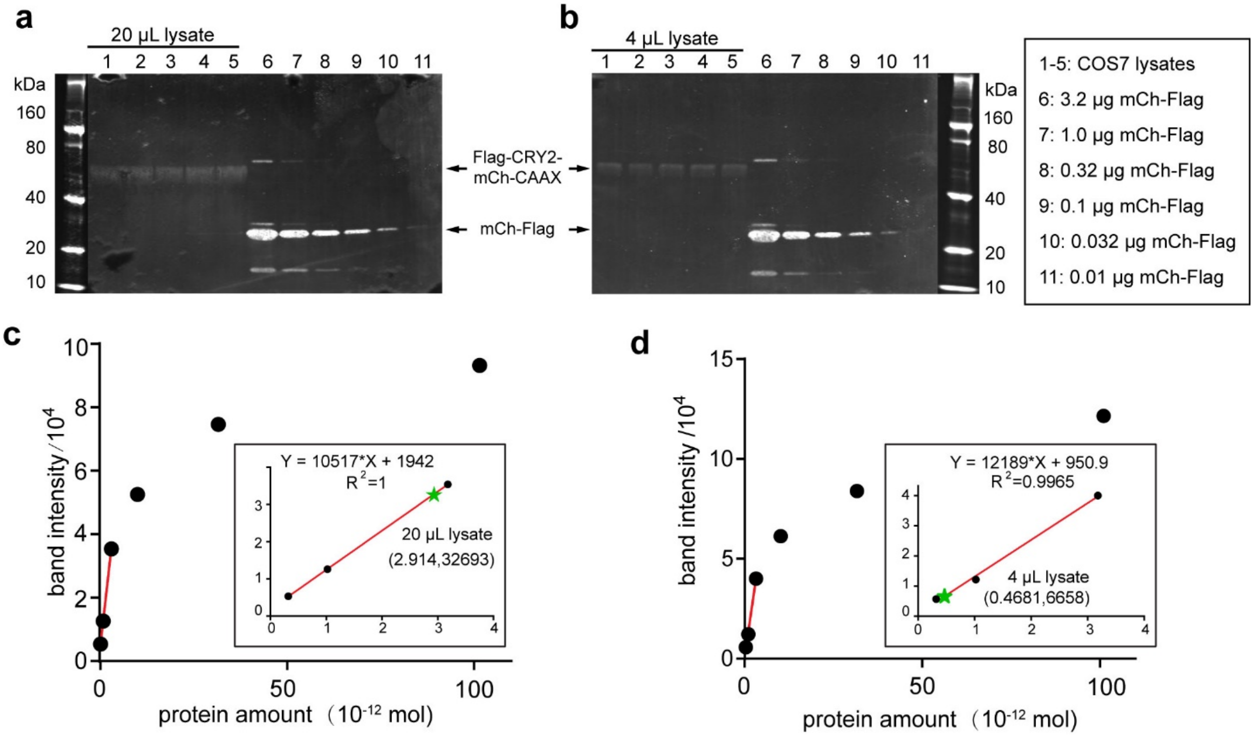

Supplementary Figure 11. Quantification of CRY2 amount in cell cultures. 5 wells of COS7 cells expressing Flag-CRY2low-mCh-CAAX were lysed in 40µL lysate. Gel was loaded with purified mCh-Flag and 20µL cell lysates (a) or 4µL cell lysates (b) and immunostained against Flag. (c) The band intensity was plotted against protein amount of purified mCh-Flag using results from blot a. (d) The band intensity was plotted against protein amount using purified mCh-Flag using results from blot b. Calibration lines (insets in c and d) were obtained using the intensities of three lowest mCh-Flag amount. The average intensities of cell lysates from 5 wells in either blot were marked in green on the calibration lines.

Supplementary Table 1. Plasmid Construction.

Plasmid Name	Method	Template	Insertion Sites	Forward Primer	Reverse Primer
CRY2(neutral2-6)-mCh	InFusion	CRY2wt-mCh	XhoI + EcoRI	ggactcagatctcgaggccaccatgtcttcaggatcatctactatagtttggtttag	tagtgaagtagaattccc
CRY2(Δ2-6)-mCh	InFusion	CRY2wt-mCh	XhoI + EcoRI	ggactcagatctcgaggccaccatgactatagtttggtttagaag	tagtgaagtagaattccc
CIB1-GFP-Sec61	Ligation	CIB1-GFP	Bsp1407+MfeI	n/a	n/a
CRY2(Δ491-498)	InFusion	CRY2wt-mCh	EcoRI + XmaI	aagttgttgggaattctacttc	accggtggatcccgggcttcacgggttcttgaaatag
CRY2(Δ490-498)	InFusion	CRY2wt-mCh	EcoRI + XmaI	aagttgttgggaattctacttc	accggtggatcccgggcacgggttcttgaaatagctttag
CRY2(Δ489-498)	InFusion	CRY2wt-mCh	EcoRI + XmaI	aagttgttgggaattctacttc	accggtggatcccgggcggttcttgaaatagctttag
CRY2(Δ488-498)	InFusion	CRY2wt-mCh	EcoRI + XmaI	aagttgttgggaattctacttc	accggtggatcccgggctcttgaaatagctttagctag
CRY2(Δ487-498)	InFusion	CRY2wt-mCh	EcoRI + XmaI	aagttgttgggaattctacttc	accggtggatcccgggctgaaatagctttagctagtag
CRY2(E490G)-mCh	InFusion	CRY2wt-mCh	EcoRI + XmaI	aagttgttgggaattctacttc	accggtggatcccgggctgctgctccgatcatgatctgtgctccacgggttcttg
CRY2(E490K)-mCh	InFusion	CRY2wt-mCh	EcoRI + XmaI	aagttgttgggaattctacttc	accggtggatcccgggctgctgctccgatcatgatctgtgctttacgggttcttg
CRY2(E490R)-mCh	InFusion	CRY2wt-mCh	EcoRI + XmaI	aagttgttgggaattctacttc	accggtggatcccgggctgctgctccgatcatgatctgtgctctacgggttcttg
CRY2(E490H)-mCh	InFusion	CRY2wt-mCh	EcoRI + XmaI	aagttgttgggaattctacttc	accggtggatcccgggctgctgctccgatcatgatctgtgcgtgacgggttcttg
CRY2 (R489D)-mCh	InFusion	CRY2wt-mCh	EcoRI + XmaI	aagttgttgggaattctacttc	accggtggatcccgggctgctgctccgatcatgatctgtgcttcatcggttcttg
CRY2 (R489E)-mCh	InFusion	CRY2wt-mCh	EcoRI + XmaI	aagttgttgggaattctacttc	accggtggatcccgggctgctgctccgatcatgatctgtgcttcttcggttcttg
CRY2 (R489E, E490R)-mCh	InFusion	CRY2wt-mCh	EcoRI + XmaI	aagttgttgggaattctacttc	accggtggatcccgggctgctgctccgatcatgatctgtgctctttcggttcttg
CRY2(1-488)E	InFusion	CRY2wt-mCh	EcoRI + XmaI	agttgttgggaattctacttc	accggtggatcccgggcctcggttcttgaaatagctttag
CRY2(1-488)EE	InFusion	CRY2wt-mCh	EcoRI + XmaI	agttgttgggaattctacttc	accggtggatcccgggcttcctcggttcttgaaatagctttagc
CRY2(1-488)EED	InFusion	CRY2wt-mCh	EcoRI + XmaI	agttgttgggaattctacttc	accggtggatcccgggcgtcttcctcggttcttgaaatagctttagc
CRY2(1-488)EEDD	InFusion	CRY2wt-mCh	EcoRI + XmaI	agttgttgggaattctacttc	accggtggatcccgggc gtcgtcttcctcggttcttgaaatag
tdTom-CIB1	InFusion	GFP-CIB1	NheI + Bsp1407	cgtcagatccgctagcgtacgacgatgacgataaggatcc	atggatcccttgtacagctcgtccatgccg
CIB1-tdTom	InFusion	CIB1-GFP	AgeI+MfeI	atattcatgtaccggtcgccaccatgatggtgagcaagggcgaggag	ttaacaacaacaattgttacttgtacagctcgtccatg
CRY2wt-tdTom	InFusion	CRY2wt-mCh	Crf9I + NotI	ggagcagcagcccgggataaggatcccgccaccatgg	tctagagtcgcggccgcttacttgtacagctcgtccatg
tdTom-CRY2wt	InFusion	mCh-CRY2	NheI+Bsp1407	cgtcagatccgctagcgtacgacgatgacgataaggatcc	ctactgcccttgtacagctcgtccatgccg
CRY2low-tdTom	Ligation	CRY2(1-488)EED	XmaI+EcoRI	n/a	n/a
CRY2high-mCh-Raf1	InFusion	CRY2wt-mCh-Raf1	EcoR1	aagttgttgggaattctacttc	cagcactatcgaattctgctgctccgatcatgatc
CRY2low-mCh-Raf1	InFusion	CRY2wt-mCh-Raf1	EcoRI	aagttgttgggaattctacttc	cagcactatcgaattcgtcttcctcggttcttgaa
CRY2low-tdTom-Raf1	Ligation	CRY2wt-mCh-Raf1	Bsp1407I+NotI	n/a	n/a
Flag-CRY2 (high, wt, low)-mCh	Ligation	CRY2 (high, wt, low)-mCh	NheI + XhoI	ctagcgccaccatggactacaaagacgatgacgacaagc	tcgagcttgtcgtcatcgtctttgtagtccatggtggcg
Myc-CRY2(high, wt, low)-mCh	Ligation	CRY2 (high, wt, low)-mCh	NheI + XhoI	ctagcgccaccatggaacaaaaactcatctcagaagaggatctgc	tcgagcagatcctcttctgagatgagtttttgttccatggtggcg
CRY2-mCh(high, wt, low)-CAAX	InFusion	CRY2 (high, wt, low)-mCh	Bsp1407I + MfeI	atggacgagctgtaca	ttaacaacaacaattgcat
Flag-CRY2low-mCh-CAAX	Ligation	CRY2low-mCh-CAAX	NheI + XhoI	ctagcgccaccatggactacaaagacgatgacgacaagc	tcgagcttgtcgtcatcgtctttgtagtccatggtggcg

Supplementary Methods

Converting intracellular fluorescence intensities to absolute concentrations

The 2-dimensional concentration of CRY2 was calculated by (the total protein amount of CRY2)/(the sum of cell areas).

1. **Quantifying the cell covering area per cell culture well**

   For membrane-bound CRY2, we calculated its 2-dimensional protein concentration using #molecules/area. We first measured the covering area of transfected cells per cell culture well in a 12-well plate. Briefly, 20 mCh images were randomly taken throughout the culture using a 20x objective. The sum of cell areas in each image was measured using ImageJ. The percentage of cell covering area in each image was quantified by (sum of cell areas)/(size of image area). For one well in 12-well plate, the sum of cell areas was calculated by (size of one well in a 12-well plate)*(percentage of cell covering area). In one well, the total area of cells is 103 mm^2.

2. **Quantifying the absolute CRY2 concentration on the cell membrane**
   We quantified the total amount of Flag-CRY2low-mCh-CAAX protein per cell culture well by using purified mCh-Flag with known concentrations as calibration standards. Briefly, 5 wells of COS7 cells expressing Flag-CRY2low-mCh-CAAX in a 12-well plate were lysed separately using preheated 40µL SDS+β-mercaptoethanol mixture (9:1 ratio). 20 µL or 4 µL lysate from each lysis was loaded in NuPAGE 4-12% Bis-Tris gel along with varied amount of purified mCh-Flag (0.01ug to 3.2µg) as calibration standards. mCh-Flag was purified from XL10-Gold competent cells expressing mCh-Flag with 6xHis tags at the N-terminus. For quantification, we did Western blot using anti-Flag antibody produced in goat (Santa Cruz Biotechnology) and IRDye 800CW conjugated anti-goat IgG produced in Donkey (LI-COR Biosciences). Fluorescence from gel was acquired by Odyssey CLx (**Supplementary Fig. 11a**: 20µL lysates, **Supplementary Fig. 11b**: 4µL lysates), and band intensities were measured using ImageJ. For both blots, the intensities of three lowest amount (0.01, 0.032, 0.1 µg mCh-Flag, equivalent to 3.18×10^{-13},1.02×10^{-12}, 3.18×10^{-12} mol of proteins) increase linearly as the protein amount increases, which were used to construct the calibration line. The intensities of cell lysates fell within this linear range, and the amount of protein in each cell lysate was calculated using the calibration line. As shown in **Supplementary Fig. 11c and d**, the 20ul lysate contains 2.91×10^{-12} mol of proteins (in blot shown in **Supplementary Fig. 11a**), while the 4ul lysate (in blot shown in **Supplementary Fig. 11b**) contains about 0.468×10^{-12} mol of Flag-CRY2low-mCh-CAAX. Therefore, the average amount of Flag-CRY2low-mCh-CAAX protein in one well of 12-well plate (40 ul lysate) was 5.25×10^{-12} mol, which corresponds to 3.16×10^{12} molecules. Therefore, the average CRY2 concentration is calculated to be 3.16×10^{12} molecules/103 mm^2 =3.07×10^4 molecules μm^{-2}.

3. Quantifying the concentration of CRY2 in individual cells.

We quantified the CRY2 concentrations in individual cells by correlating CRY2 protein concentration with mCh fluorescence intensity. We first measured the average fluorescence intensity of Flag-CRY2low-mCh-CAAX. The mCh images of 630 cells expressing Flag-CRY2low-mCh-CAAX were randomly acquired throughout the culture using a 100x objective. The mCh intensity of each cell was then measured using ImageJ and calculated as (average intensity of cell - image background intensity). The average fluorescence intensity of 630 cells was measured as 1900, which would correspond to the average CRY2 concentration/area of 3.07×10^4 molecules μm^{-2}. CRY2 concentrations in individual cells were calculated by linearly scaling with the fluorescence intensities.
